# Quality of Blood Pressure Tracking Apps for the iPhone: Content Analysis and Evaluation of Adherence With Home Blood Pressure Measurement Best Practices

**DOI:** 10.2196/10809

**Published:** 2019-04-12

**Authors:** Amanda Y Leong, Mark J Makowsky

**Affiliations:** 1 Faculty of Pharmacy & Pharmaceutical Sciences University of Alberta Edmonton, AB Canada; 2 Saskatchewan Health Authority, Regina Region Regina, SK Canada

**Keywords:** hypertension, mobile apps, self-management, blood pressure monitoring, ambulatory, cross-sectional studies

## Abstract

**Background:**

Blood pressure (BP) tracking apps may aid in hypertension (HTN) self-management, but app quality may be problematic.

**Objective:**

This study aimed to develop a content-dependent rating system for BP tracking apps and systematically evaluate BP tracking features, content-independent quality, functional characteristics, and educational comprehensiveness of English language iPhone apps developed with the primary purpose of tracking a consumer’s BP measurements.

**Methods:**

We created a 28-item checklist reflecting overall app quality and a simplified 2-item checklist to assess adherence with home BP monitoring best practices. Apps with educational information were evaluated for comprehensiveness on a 7-point scale and for consistency with evidence-based guidelines. Higher scores represent better quality and comprehensiveness. We searched the Canadian App Store on June 28, 2016, using the keywords *hypertension* and *blood pressure*. A total of 2 reviewers independently assessed apps according to the standardized template. We determined if paid apps, educational apps, or those rated ≥4 stars were of higher quality.

**Results:**

Of the 948 apps screened, 62 met the inclusion criteria. The mean overall quality score was 12.2 (SD 4.6, out of 28) and 6 apps (10%, 6/62) met the home BP monitoring best practice criteria. In all, 12 apps contained educational content (mean comprehensiveness 2.4, SD 1.6 out of 14), most commonly, background information on HTN. Apps with educational content (mean 15.1, SD 3.8 vs 11.8, SD 4.8; *P*=.03) or a ≥4 star rating (median 19, interquartile range [IQR] 15-20, vs 12, IQR 9-15; *P*=.02) had higher overall quality.

**Conclusions:**

The BP tracking apps reviewed had variable quality and few met the home BP monitoring best practice criteria. When deciding to recommend a specific BP tracking app, we suggest clinicians should evaluate whether the app allows input of duplicate BP readings in the morning and evening for at least seven days and presents the mean BP value for user-specified dates. Greater attention to home BP measurement best practices is required during app development.

## Introduction

### Background

High blood pressure (BP) affects approximately 31% of adults globally [1] and affects 23% of Canadian adults [2]. In 2010, hypertension (HTN) was the leading cause of death and disability-adjusted life years worldwide; in 2015, high systolic BP accounted for 10.7 million deaths and 211.8 million disability-adjusted life years [3,4]. Lifestyle modification and medication management effectively reduce cardiovascular risk in patients with HTN. For example, a multicomponent intervention promoting increased physical activity, weight loss, reduced sodium intake, and the Dietary Approaches to Stop Hypertension (DASH) diet, reduced the HTN prevalence in those with above optimal BP at 18 months compared with those receiving advice alone—22% versus 32%; odds ratio, OR 0.77 (95% CI 0.62-0.97) [5]. Antihypertensive pharmacotherapy reduces the relative risk of myocardial infarction by 20% to 25%, stroke by 30% to 40%, and heart failure by nearly 50% [6].

Guidelines consistently recommend regular home BP monitoring for HTN management, particularly in patients with established HTN, comorbid diabetes or chronic kidney disease, suspected nonadherence, and white coat or masked HTN [7-10]. Although these recommendations are based on weak evidence and expert opinion, a recent systematic review and meta-analysis found that home BP monitoring for 6 months leads to a significant decline in systolic blood pressure (SBP) by 3.9 and diastolic blood pressure (DBP) by 2.4 mmHg versus usual care [11]. Home BP telemonitoring is also associated with larger reductions in office (SBP 4.7 mmHg, DBP 2.5 mmHg) and ambulatory BP versus usual care [12]. Recent guidelines suggest that home BP monitoring may also be used in the diagnosis of HTN [10,13-15].

Health interventions using mobile technology, that is, mobile health (mHealth), are increasingly used to provide patients and health care professionals with additional tools and resources to manage chronic disease [16], including HTN [17-20]. For example, a systematic review found that digital health interventions significantly reduced cardiovascular disease outcomes in primary and secondary prevention populations (relative risk 0.61; 95% CI 0.46-0.80, I^2^=22%) but had no influence on SBP (−1.18 mmHg, 95% CI −2.93 mmHg to 0.57 mmHg, I^2^=100%) [18]. In contrast, a small study using a mobile-based self-management support system significantly reduced systolic (−7 mmHg) and diastolic (−4.9 mmHg) BP over 8 weeks [21]. Furthermore, a mobile phone–based medication reminder app improved adherence and BP among patients with HTN [19]. Therefore, mHealth apps may serve to enhance BP control in patients with HTN by providing a flexible, convenient platform for patient self-management.

### Rationale and Objective

Assessing the content and quality of medical apps designed for consumers may help clinicians recommend reliable and accurate apps as well as promote safe app use by patients, but only 1 published study has previously evaluated HTN apps [22-25]. In 2014, Kumar et al conducted a content analysis of the functional characteristics and consumer interaction metrics for the top 107 HTN-related apps for Apple iPhone and Google Android devices [25]. They reported that a majority of apps are designed to track BP, weight, or body mass index and concluded that greater oversight is needed in medical HTN app development, especially apps qualifying as medical devices. However, they did not conduct a formal evaluation of the quality and usefulness of the BP tracking functionalities, for example, whether available apps allow tracking of duplicate home measurements every morning and evening over a 7-day period with calculation of mean BP excluding the first day readings for clinical decision making as recommended by experts [15,26-29].

To address this gap and assist both clinicians and patients in selecting high-quality apps that could be used in the diagnosis and management of HTN, our objective was to develop a content-dependent rating system for BP tracking apps and to systematically evaluate the BP tracking features, content-independent quality, functional characteristics, and educational comprehensiveness of currently available English language iPhone apps developed with the primary purpose of tracking a consumer’s home BP measurements.

## Methods

### Criteria for Assessment of Blood Pressure Tracking Apps

We used several international HTN clinical practice guidelines statements [26-29] and the systematic review of asthma self-management apps by Huckvale et al to guide development of criteria and domains for our BP tracking app evaluation tool (Table 1; Multimedia Appendix 1) [30,31].

**Table 1:** Features of the reviewed apps (N=62).

Demographics and features			Statistic
**Free, n (%)**			38 (61)
	No option to upgrade		20 (53)
	Option to upgrade		18 (47)
**Paid only, mean (SD)**			24 (39)
	Average cost, mean (SD)		Can $2.54 (1.10)
**App store category, n (%)**			
	Medical		34 (55)
	Health & fitness		28 (45)
	Presence of a star rating		12 (19)
	≥4 stars		5 (8)
**Country of origin, n (%)**			
	United States of America		14 (23)
	Germany		8 (13)
	Unclear		28 (45)
	Other		12 (19)
**Content rating, n (%)**			
	4+		34 (55)
	12+		18 (29)
	17+		10 (16)
**Sponsored and created by, n (%)**			
	Software company		34 (55)
	Pharmaceutical company		1 (2)
	Medical/device company		6 (10)
	Health organization		1 (2)
	Individual person		20 (32)
App can transform the phone into a medical device			0 (0)
**BP^a^ tracking features, n (%)**			
	Backdate BP measurements		53 (86)
	Duplicate measures QAM^b^ and QHS^c^ for 7 days		49 (79)
	Personal notes or diary for each BP reading		39 (63)
	Reminders		20 (32)
	BP goal-setting		12 (19)
	Sync BP data with a BP monitor		12 (19)
**Data validation, n (%)**			
	Rational BP max & min limits		29 (47)
	Flags inverted readings		16 (26)
**BP analytical features, n (%)**			
	BP measurements are categorized		41 (66)
	Appropriate course of action suggested in alert ranges		5 (8)
	In-app graphing of BP measurements		55 (89)
	In-app statistical analysis (overall)		36 (58)
	In-app statistical analysis (unchangeable pre-set dates)		30 (48)
	In-app statistical analysis (user-specified dates)		6 (10)
Data export, n (%)			50 (81)
Live sharing, n (%)			1 (2)
Cloud-based data backup, n (%)			9 (15)
**HON^d^ quality**			
	1. Health care professional involvement		4 (7)
	2. Disclaimer—not a replacement for a health care provider		27 (44)
	3. Privacy policy present		28 (45)
	4a. App updated in past year		28 (45)
	**4b. BP categories have reference to scientific research**		
		BP categories not present	22 (36)
		BP categories not referenced	31 (50)
		BP categories referenced	9 (15)
	5. Contact details for support		33 (55)
	6. Conflict of interest or sponsorship clearly labeled		28 (45)
	7. Advertising clearly distinguishable from content		12 (19)
**Expanded HON criteria**			
	No advertising		47 (76)
	Promotes a specific product		5 (8)
**Functional characteristics**			
	BP-tracking		62 (100)
	Heart rate tracking		56 (90)
	Weight tracking		21 (34)
	Medication tracking		11 (18)
	Built in educational content		11 (18)
	Exercise tracking		8 (13)
	Diet tracking		4 (7)
	Symptom tracking		3 (5)
	Social media		4 (7)
	Salt tracking		3 (5)
	Lab values tracking		1 (2)
	Referral to outside education resources		1 (2)
	Cardiovascular risk calculator		0 (0)

^a^BP: blood pressure.

^b^QAM: each morning.

^c^QHS: at night.

^d^HON: Health on the Net.

#### Blood Pressure Tracking Features

BP tracking features were evaluated in 6 areas regarding the following abilities: to record duplicate morning and evening measurements for at least one week, set a BP goal, set reminders to take BP readings, sync data with a home BP monitor, backdate and time stamp readings, and log personal notes with each reading.

#### Blood Pressure Data Validation

BP pressure data validation was assessed using 2 items: presence of rational minimum and maximum BP limits (scrolled data entry) or warning about improbably low or high readings (typed in readings), and warning about inverted entry of systolic and diastolic readings.

#### Blood Pressure Analytical Features

BP analytical features were assessed in 7 areas regarding the following abilities: to conduct in-app statistical analysis including mean BP readings, perform in-app graphing of BP measurements, categorize BP readings and red flag abnormal readings, suggest an appropriate course of action for readings in alert ranges, export data for sharing with others, automatically share readings, and perform cloud-based data backup. The app evaluation tool awarded 1 point for positive responses and 0 points for negative responses, for most items. Some items were scaled on 2-, 3-, or 4-point scales according to the level of functionality present (Multimedia Appendix 1).

#### Content Independent Health on the Net Quality

The quality of all apps was assessed using modified content-independent criteria created by the Health on the Net (HON) Foundation and used by Huckvale et al [30-32]. The 8 items were adapted to focus on BP tracking apps and included whether or not the app included a health care professional, as defined by the Health Professions Act on the authorship and development team, a clear purpose or disclaimer that it is not meant to replace the advice of a health care professional, a privacy policy, a recent update (ie, an update in the past 12 months), BP categories referenced using scientific literature, contact information for the app developers, a sponsorship statement and clear labeling of sponsors, and finally, a clear distinction between advertising, if present, and content. One point was awarded for positive responses and 0 points were awarded for negative responses.

#### Functional Characteristics

Functional characteristics were rated according to 13 items including the presence of a BP log, heart rate log, symptoms log, and cardiovascular risk calculator; trackers for exercise, diet, dietary sodium, weight, medication, and lab values (eg, sodium, potassium, serum creatinine); compatibility with social media platforms and presence of built-in educational material or referral to outside resources for HTN education.

### Apps Containing Educational Material

For apps that combined BP logging features with health information, the information in these apps was assessed in 2 domains: comprehensiveness and consistency with evidence-based guidelines.

#### Comprehensiveness

We assessed 7 topics, whether the app contained information on BP basics, treatment options, how to use treatments, BP self-monitoring technique, a personalized action plan, how to recognize abnormal BP values, and links to health care providers. For each topic, coverage was assessed as Present in entirety (2 points), Partial (1 point), or Not present (zero points).

#### Consistency

We extracted key messages that were consistent among 3 international guidelines regarding BP measurement techniques (7 items) and lifestyle management (6 items) [14,26,33]. One point was awarded for information consistency and 0 points for information inconsistency, with selected statements.

#### Home Blood Pressure Monitoring Best Practices

Finally, given the complexity of the proposed 28-item evaluation tool, we evaluated each app against a 2-item home BP monitoring best practices criterion as suggested in major clinical practice guidelines [27-29]. Apps that allowed input of duplicate BP readings in the am and pm for at least seven days and contained in-app statistical analysis, allowing for calculation of mean BP values on user-specified dates, were deemed to meet this criterion [26-29].

A mock patient and predefined set of tasks was developed to ensure consistent, comprehensive evaluation of each app (Multimedia Appendix 2). Our full assessment form went through several iterations and rounds of testing before it was fully implemented in the review.

### Search Strategy

In total, 2 search terms, *hypertension* and *blood pressure*, were used to identify English language apps focused on tracking serial BP measurements for adult members of the general public or HTN patients available in the Canadian Apple App Store. A preliminary search was conducted between May 22, 2015, and July 28, 2015, (AL) using an Apple iPad model MC769C/A, iOS version 8.4, and the final search was conducted on June 28, 2016, by a second investigator (MJM) using an Apple iPhone 7, iOS version 10.3.2, from Edmonton, Alberta, Canada.

### App Selection and Data Extraction

Apps were included if the title or the app description indicated that the primary function of the app was to track BP measurements over time, the app was intended for use by the general public or HTN patients, was in English, and available in Canada. Preliminary searching indicated a large number of potentially relevant apps. Rather than limit our search to the top 50 apps as done by others [25], we narrowed the scope of the review to apps that focused on BP tracking for uncomplicated HTN. Therefore, we excluded apps advertised as whole health trackers where BP was only 1 of several tracked parameters and those that focused on HTN in the context of comorbid diabetes, chronic kidney disease, or other chronic health conditions. We excluded apps that required the purchase of a proprietary BP monitor as the only means to enter BP readings into the app. Both paid and free apps were analyzed, but when both free and paid versions were available, only free versions of apps were analyzed. Finally, we excluded apps that contained 2 or more technical or functional errors that made the app unusable and apps costing more than Can $19.99.

App name, developer, and cost were extracted for all apps by a single reviewer (MJM). Subsequently, a 2-phase screening process was completed independently by both reviewers. The first screen was based on the information provided in the App Store summary and any linked webpages. The second screen was conducted on the basis of the information in the app after it was downloaded. In cases of disagreement, a third party was asked to assess the App Store summary, and an agreement was reached by consensus. All apps that passed the screening process had the following descriptive information recorded from the App Store: category, date of last update, version, parental rating, original release date, current version, and average user star rating.

### Statistical Analysis

We calculated the scores for each respective domain by summing the individual response scores for each component. The usefulness of an app’s BP tracking functionality was calculated as the sum of the BP tracking features, BP data validation, and BP analytical features domain scores. We summed the usefulness score with the HON quality score to create the app overall quality score.

We analyzed measures of central tendency for all variables and scores. Parametric data were presented as mean (SD), whereas nonparametric data were presented as median (interquartile range, IQR). We explored whether paid apps were of higher quality than free apps, whether apps with an educational component were of higher quality than those without an educational component, and whether apps with user star ratings ≥4 were of higher overall quality than those rated less than 4 stars or without a rating. Two-tailed Mann Whitney U or 2 independent sample *t* tests were used as appropriate to compare median domain scores and mean overall quality scores between groups. All data were extracted to Microsoft Excel for Mac 2011 (Microsoft Corp), and statistical analysis was conducted using SPSS (IBM SPSS Statistics for Mac, Version 24.0, IBM Corp).

## Results

### Characteristics of Included Apps

**Figure 1:**
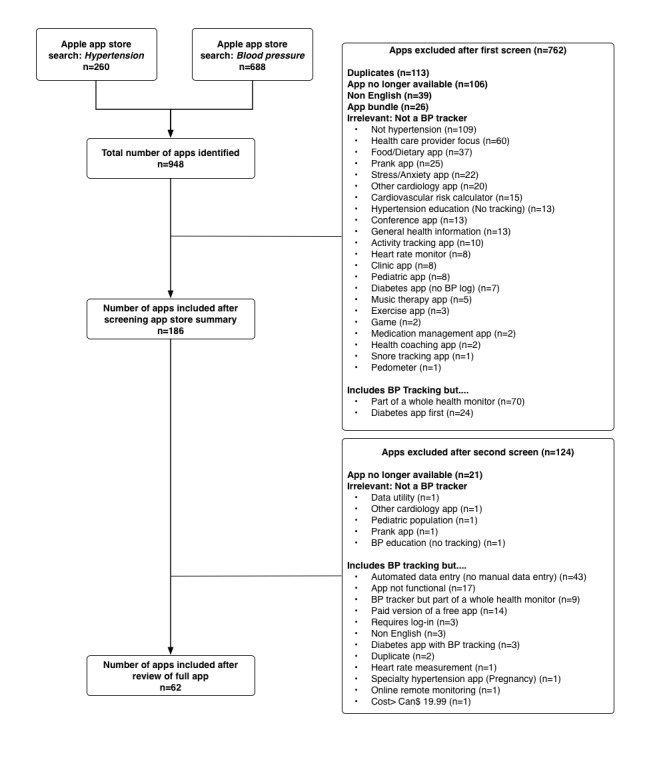
Flow diagram. BP: blood pressure.

Of the 948 apps screened, 62 (6.5%) met the inclusion criteria (Figure 1). A majority of apps were excluded as they were deemed not relevant to HTN tracking (41.0%; 389/948), they were no longer available for download when the assessment started in May 2017 (13.4%; 127/948), or they were duplicate records (12.1%; 115/948). Of note, several apps with BP tracking functionality were excluded (n=79 whole health monitors; n=43, which required automated transmission of data from a BP monitor; n=27, which were diabetes apps centered on blood glucose tracking; n=17, which were no longer functional; n=1, which cost more than Can $19.99; Figure 1).

Characteristics of the included apps are shown in Table 1. In total, 24 apps required payment and had an average cost of Can $2.54 (SD 1.10). Of the 38 free apps, 18 had an option to upgrade to a paid version/offered in-app purchases. All included apps were categorized as medical or health and fitness apps. Only 19% (12/62) of included apps had a user rating for the current version, and only 8% (5/62) were rated as ≥4 stars. The identified apps were most commonly created in the United States (23%; 14/62) and Germany (13%; 8/62). Most apps were sponsored by/created by a software company (53%; 34/62), an individual person (33%; 20/62), or a medical device company (9%; 6/62). A list of all the included apps is available in Multimedia Appendix 3.

### Overall Quality Score and Adherence With Home Blood Pressure Monitoring Best Practice Criteria

The mean overall quality score was 12.5 (SD 4.8) out of 28, and it ranged from 2 to 22 (Table 2). In total, 10% (6/62) of included apps met the proposed home BP monitoring best practice criteria.

### Usefulness of Blood Pressure Tracking Functions

The mean BP tracking usefulness score was 9.7 (SD 3.7) out of 20 **(**Table 2). The mean BP tracking features domain score was 3.7 (SD 1.6) out of 7, and as shown in Table 1, most apps allowed for the entry of duplicate measures in the morning/evening for 7 days (79%; 49/62), or they allowed backdating of inputted measures (86%; 53/62), whereas a few allowed BP goal setting (19%; 12/62), reminders (32%; 20/62), or allowed automated entry of BP readings (19%; 12/62). The mean data validation domain score was 0.7 (SD 0.7; Table 2) and 47% (29/62) had rational maximum and minimum BP limits, whereas only 26% (16/62) flagged inverted systolic and diastolic BP readings. The mean analytic features domain was 5.3 (SD 2.3; Table 2). Although 58% (36/62) of the apps allowed in-app analysis of BP readings, only 10% (6/62) allowed for in-app calculation of average BP readings using user dates (Table 1). Most allowed for in-app graphing of BP readings (89%; 55/62), data export (81%; 50/62), or categorization of BP measurements (66%; 41/62), whereas only 8% (5/62) suggested an appropriate course of action when BP readings were in alert ranges.

**Table 2:** Hypertension app scores for functionality, usefulness, and quality (n=62).

Domain		Potential score range	Mean (SD)	Median (IQR^a^)	Minimum	Maximum
**Overall quality score^b^**		0-28	12.5 (4.8)	12 (9-16)	2	22
	BP^c^ tracking usefulness score	0-20	9.7 (3.7)	10 (8-13)	2	18
	BP tracking features	0-7	3.7 (1.6)	4 (2-5)	1	7
	Data validation	0-2	0.7 (0.7)	1 (0-1)	0	2
	BP analytical features	0-11	5.3 (2.3)	6 (4-7)	0	10
	Health on the Net quality	0-8	2.8 (1.7)	2 (1-4)	0	6
Functional characteristics		0-13	3.0 (1.5)	2.5 (2-4)	1	9
Education comprehensiveness (n=12)		0-14	2.4 (1.6)	2 (1-3)	0	6
BP measurement content (n=3)		0-7	3.3 (1.2)	4 (3-4)	2	4
Lifestyle content (n=7)		0-6	1.7 (1.4)	2 (0-3)	0	3

^a^IQR: interquartile range.

^b^Overall quality score: BP tracking features, data validation, BP analytical features, and Health on the Net quality.

^c^BP: blood pressure.

### Health on the Net Quality Scores

The median HON quality score was low (2, IQR 1-4 out of 8, Table 2). As shown in Table 1, just under half (45%; 28/62) of the apps were updated in the past year, contained a privacy policy (44%; 28/62), had contact details or support (55%; 33/62), and had clearly labeled sponsorships (45%; 28/62). A minority had health care professional involvement, a disclaimer, and references to scientific research for BP ranges. Only 15% (9/62) referenced scientific literature for the BP category cut-offs used.

### Functional Characteristics

The median number of functional characteristics was 2.5 (IQR 2-4, Table 2). Most apps had at least one additional feature beyond BP and heart rate tracking (53%; 33/62). Most common were weight tracking (34%; 21/62), educational content (18%; 11/62), or medication tracking (18%; 11/62; Table 1). Although 1 app had 9 different functions, few apps (26%; 16/62) had 4 or more built-in functions.

### Apps Containing Educational Material

The median educational comprehensiveness in the 12 apps that contained educational content was 2 (IQR 1.5-2) out of 14 (Table 2)**.** Most apps contained basic information about BP and HTN (75%; 9/12), lifestyle management options (58%; 7/12), and encouraged sharing of BP readings with health care professionals (42%; 5/12), whereas few (25%; 3/12) provided information about BP measurement technique (Table 3).

**Table 3:** Assessment of apps containing educational content.

Features		Statistics, n (%)
**Comprehensiveness (n=12)**		
	Basic BP^a^ and hypertension information	9 (75)
	Lifestyle treatment options	7 (58)
	How to use treatment	2 (17)
	BP self-monitoring technique	3 (25)
	Personalized action plan (HBPM^b^ treatment goal <135/85 mmHg)	0 (0)
	Recognition of abnormally high or low BP values	2 (17)
	Link to health care provider	5 (42)
**BP measurement (n=3)**		
	Wait 30 min after coffee or smoking	3 (100)
	Relax before BP reading	3 (100)
	Body positioning	2 (67)
	Consistently measure BP the same arm	2 (67)
	Validated BP device	0 (0)
	BP measured ≥2 times QAM^c^ & QHS^d^ ≥7 days	0 (0)
	Arm automatic monitor with well fitted cuff	0 (0)
**Lifestyle content (n=7)**		
	Advice to quit smoking	4 (57)
	Exercise	3 (43)
	Body mass index or waist circumference targets	0 (0)
	Alcohol restriction	1 (14)
	Heart healthy diet	3 (43)
	Goal daily sodium: 2 g	1 (14)
	Written in plain language	10 (83)
	Grammatical and spelling errors present	3 (25)

^a^BP: blood pressure.

^b^HBPM: home-blood pressure measurement.

^c^QAM: each morning.

^d^QHS: at night.

### Exploring Influence of Cost, Educational Content, and User Rating

There were no statistically significant differences in functional characteristics, usefulness, HON quality, or overall quality between free and paid apps (Table 4). Apps with an educational component had higher overall quality scores than those without an educational component (mean 15.1, SD 3.8 vs 11.8, SD 4.8; *P*=.03). Although only 5 apps were rated ≥4 stars, those with this rating had a higher overall quality score (median 19, IQR 15-20, vs 12, IQR 9-15; *P*=.02). These appeared to be driven by both higher usefulness and HON quality scores.

**Table 4:** Comparison of hypertension app domain and overall quality scores by cost, presence of educational content, and user rating (n=62).

Domain	Free (n=38)	Paid (n=24)	*P* value	No education (n=50)	Education (n=12)	*P* value	<4 stars (n=57)	≥4 stars (n=5)	*P* value
Overall quality score	12.7 (5.2)^a^	12.1 (4.0)^a^	.63	11.8 (4.8)^a^	15.1 (3.8)^a^	.03	12 (9-15)^b^	19 (15-20)^b^	.02
BP^c^ tracking usefulness score	9.8 (4.1)^a^	9.5 (3.1)^a^	.71	9.2 (3.7)^a^	11.5 (3.0)^a^	.06	10 (7-12)^b^	13 (11-15)^b^	.04
BP tracking features, median (IQR^d^)	4 (2-5)	4 (3-4)	.41	4 (2-5)	4 (3-5)	.32	4 (2-5)	5 (4-6)	.12
Data validation. median (IQR)	1 (0-1)	1 (0-1)	.55	1 (0-1)	1 (0-2)	.20	1 (0-1)	1 (1-2)	.47
BP analytical features, median (IQR)	6 (4-7)	5.5 (4-7)	.80	5 (3-7)	7 (5-7)	.09	5 (4-7)	7 (6-8)	.05
HON^e^ quality, median (IQR)	2.5 (1-5)	2 (2-4)	.73	2 (1-4)	3.5 (2-5)	.07	2 (1-4)	5 (4-6)	.01
Functional characteristics, median (IQR)	3 (2-4)	2 (2-3)	.44	2 (2-3)	3.5 (3-7)	<.001	2 (2-4)	3 (3-5)	.22
Education comprehensiveness, median (IQR)	2 (2-5)	2 (1-2)	.20	—^f^	—	—	—	—	—

^a^Data are presented as mean (SD).

^b^Data are presented as median (IQR). Aggregate BP usefulness and overall quality scores were normally distributed for each comparison group except by 4-star rating. All single-item domain scores were not normally distributed.

^c^BP: blood pressure.

^d^IQR: interquartile range.

^e^HON: Health on the Net.

^f^Not applicable.

## Discussion

### Principal Findings

In this review, we found concerning gaps in BP tracking features and large variation in the overall quality of the 62 reviewed apps according to the evaluation tool that we developed. In total, only 6 apps met the home BP measurement best practice criteria, and although most apps allowed the entry of duplicate BP measures in the morning and evening, most did not allow for in-app statistical analysis on the basis of user-specified rather than preset fixed dates based on the date of accessing the app. Of note, no apps automatically calculated the mean BP value on the basis of the last 6 days of readings, as recommended by some organizations, and only apps that allowed users to specify the dates included allowed this calculation. Few apps flagged readings that were incorrectly input as diastolic over systolic, or they suggested an appropriate course of action when BP readings were in alert ranges. In addition, most apps used reference BP ranges set in the Joint National Committee 6 or 7 reports on HTN, which were based on office readings and none were based on the new 2017 American College of Cardiology/American Heart Association hypertension guideline [7,15]. Although some apps allowed for customization of BP target values, the treatment targets were defined on the basis of in-office measures. The content-independent HON quality was low, with the majority scoring poorly on these criteria. None of the included apps included the ability to turn the iPhone into a medical device, and although 6 apps were designed for use with a specific brand of BP monitor, none of the reviewed apps connected with a Hypertension Canada-endorsed BP monitor.

Few of the reviewed apps contained educational content, and in those that did, the materials were not comprehensive. Educational material generally focused on the basic BP background information or treatment options. Most apps were lacking key information on BP measurement technique, and none advocated the use of validated BP measurement devices, duplicate readings twice per day for 7 days, and use of an appropriately sized cuff.

We found that apps with an educational component or an App Store rating ≥4 stars were of higher overall quality compared with those without an educational component or ≤3 stars. Despite this, only 5 out of the top 10 overall quality apps and only 1 of the 6 apps meeting the best practice criteria had star ratings. On the basis of this, we suggest the use of a simplified ranking system that is not only primarily based on consistency with home BP monitoring best practice criteria but also takes into consideration the presence of educational material on BP measurement and the App Store rating (Textbox 1). Such a tool may be directly helpful for clinicians in making recommendations to patients or others regarding BP tracking apps.

**Proposed simplified, content-dependent criteria to evaluate blood pressure-tracking app quality.**

Review the app store description, screenshots, and download if necessary.

- Does the app conform to the recognized home blood pressure (BP) best practice criteria? [15,27-29]
  - Does the app allow input of duplicate BP readings for at least seven days?
  - Does the app contain *in-app* statistical analysis, which displays the mean BP values on user-specified dates?
- Does the app contain educational material on BP measurement technique or BP in general?
- Is the app rated 4 or more stars?

### Strengths and Limitations

Our study has several strengths, including a comprehensive search to identify all relevant BP tracking apps for the iOS platform, a rigorous app assessment process based on best practices from the app evaluation literature [22], and a duplicate review of all apps by independent investigators [22]. Despite the strengths, there are several limitations. First, we did not review BP tracking apps designed for the Android platform; therefore, we are potentially missing a significant number of unique apps developed exclusively for this platform. Data from 2017 suggest that approximately 46% and 43% of the smartphone market share was held by Android devices in Canada and the United States, respectively [34]. Although Kumar et al found no overlap in the top 5 most popular HTN apps by the number of downloads for iPhone and Android, there was a large degree of overlap in apps identified in Apple iTunes for iPhone and Google Play Store [25]. Second, we could only access the Canadian Apple App Store, and therefore we may not have captured apps available in other countries. Despite this, our review is applicable to clinicians internationally as we have provided both a detailed and simplified content-dependent app ranking system, the latter of which is directly applicable to clinicians in recommending useful home BP monitoring apps, regardless of the country. Third, by excluding apps that absolutely required automated transmission of data from a *smart* BP monitor to populate data into the app (eg, those from Withings, Qardioarm, and iHealth), excluding apps that were focusing on comorbid chronic conditions or were whole-health monitors, our results primarily reflect a specific subset of manual BP tracking apps available in the iOS market before the widespread availably of *smart* BP monitors. Automated *smart* BP monitors that have the ability to automatically populate data from a connected BP monitor into an app may score higher on our assessments. Although multimorbidity is common in primary care, and patients with HTN commonly have other manifestations of coronary heart disease or other chronic conditions [35], we feel justified in excluding these types of apps, as it was felt that consumers wanting to find an app to track their BP would preferentially pick 1 that did so as its primary function. In addition, by excluding apps focused on diabetes, we avoided issues associated with the controversy surrounding BP treatment targets [36]. Although we did not see major differences in paid and free apps, limiting our review to free versions of apps with a paid version may have artificially biased the usefulness and quality scores in a downward direction. Fourth, the recommendations for evaluation of mobile phone apps continue to evolve [23], and we did not use newer validated app-rating scales such as the Mobile Application Rating Scale [37]. However, our 2-item assessment tool using home BP monitoring best practice criteria was based on expert consensus recommendations from major organizations like Hypertension Canada and the American College of Cardiology/American Heart Association, which advocate for recording duplicate BP measures twice daily for at least seven days and using the mean home BP value to diagnose HTN in relation to a cut-off SBP ≥135 mmHg or DBP ≥85 mmHg [15,26,38]. Fifth, given the dynamic nature of apps in terms of content, updates, and changes in availability, the overall app quality scores and any recommendations regarding the use of specific apps are subject to change as apps are updated, new apps are released, and others are removed. Finally, by not involving patients in the app review, important components that may impact effective app use and usability (ie, limited health or technological literacy) were not systematically assessed in our review.

### Comparison With Earlier Work

The only other review of HTN self-management apps was performed by Kumar et al, who performed a content analysis of the top 50 apps for HTN for Android and iOS devices [25]. They found that 72% contained a tracking function, 22% had tools to enhance medication adherence, 37% contained general information on HTN, and 8% contained information on the DASH diet. In addition, they found that only 3% of the apps were developed by health care agencies (ie, universities or professional organizations). In contrast, our review only focused on patient-oriented apps that contained BP tracking functions and found that in these apps, 35% contained adherence tools and 18% had built-in educational content. We also found that only a minority of available apps were developed by health care agencies. Kumar et al found that the ability of apps to track BP was significantly associated with the number of app downloads. We did not explore this relationship as Apple does not release iOS app download statistics.

Our finding of a broad variation in the app quality is consistent with previous reviews of health-related mobile phone apps in the areas of diabetes, smoking cessation, pain and pulmonary management. Demidowich et al found that only 4 of 42 Android apps targeted toward diabetes self-management had sufficiently high composite usability scores, which suggested that few apps provided a comprehensive method of diabetes management [39]. In our study, only 7 apps had overall quality scores ≥19 and only 6 met the best practices criteria. Our adapted HON quality score results are similar to those of Huckvale et al, who found a generally low quality of asthma self-management apps using the HON criteria [30]. In our data, it appeared as though user star ratings may be helpful to identify a quality app, but these data are not robust, and others have found poor correlation between user-star ratings and app usability scores [39].

This study found only 12 apps that contained any sort of educational component, with no app scoring above 50% for educational quality. This demonstrates that these apps were primarily designed to be tracking tools, with little emphasis on comprehensive, high-quality educational material. Similarly, Abroms et al documented that few smoking cessation iPhone apps adhered to key guidelines and provided recommendations or linked users to proven treatments, such as pharmacotherapy, counseling, and/or quit lines [40]. Huckvale et al evaluated 103 asthma self-management apps, 38 of which contained an educational component [30]. Similar to their study, no HTN app addressed all aspects of the guidelines. In their updated 2015 study just over half (57%; 83 of 147) of apps provided educational information about asthma [31]. Therefore, it appears that asthma apps have more robust, evidence-based chronic disease self-management information than apps designed for BP tracking.

### Conclusions

A handful of apps explicitly developed and marketed for BP tracking are adherent to home BP monitoring best practices, as set out by clinical practice guidelines, and score highly on overall quality. However, several concerning gaps exist in the current BP tracking apps. Although app store ratings and the presence of educational content may help clinicians or patients choose higher quality apps, many high-quality apps did not have consumer ratings. At minimum, we suggest clinicians should evaluate whether a BP tracking app allows input of duplicate BP readings for at least seven days and presents the mean BP value for user-specified dates. There remain opportunities to improve the overall quality of patient-focused BP tracking apps and incorporate evidence-based HTN education to further optimize patient self-management of HTN.

## Appendix

Multimedia Appendix 1 Criteria for app assessment.

Multimedia Appendix 2 Mock patient used for app evaluation.

Multimedia Appendix 3 List of included apps.

## Abbreviations

BP: blood pressure
DASH: Dietary Approaches to Stop Hypertension
DBP: diastolic blood pressure
HON: Health on the Net
HTN: hypertension
IQR: interquartile range
mHealth: mobile health
SBP: systolic blood pressure
