# Supplementary material for: Quality of Blood Pressure Tracking Apps for the iPhone: Content Analysis and Evaluation of Adherence With Home Blood Pressure Measurement Best Practices
Source: JMIR Mhealth Uhealth. 2019 Apr 12;7(4):e10809. doi: 10.2196/10809 (PMC6484262; doi:10.2196/10809)
Supplement: Multimedia Appendix 1 [file mhealth_v7i4e10809_app1.pdf]

Multimedia Appendix 1. Criteria for App Assessment

Section	Subcategory	Description	Source
**Basic Characteristics**	App Name		App Store
	Free/Paid	If the app is free or paid	App Store
	Cost	Cost of the app	App Store
	Upgrade to Premium	Whether there is a premium version of the app	App Store
	Developer	The developer, either listed under App Name in the App Store, or under Seller. If there is a discrepancy, the Developer is the one whose website is listed.	App Store
	App Store Category	As listed in "app Store"	App Store
	Version	Version of the app that was studied	App Store
	Platform	Operating system: iPhone, Android, Windows, Blackberry	App Store
	Average User Rating	From the "Ratings" section in the App Store	App Store
	Targeted User	As implied by the app description in the App Store	App Store
	PG Rating	Parental content rating	App Store
	Country of Origin	Country in which the app developer is registered	App Store or Website
**Currency / Timeliness**	Original Release Date	Under the "Version History" in the App Store	App Store
	Updated in the past 1y	Are there updates listed in the "Version History" from the past 12m?	App Store
**Functional Characteristics of the App**	BP Log Present	A method of regularly recording BP values	App
	HR Log Present	A method of regularly recording HR values	App
	CV Risk Calculator Present	A risk calculator that automatically calculates the risk of CV events, such as Framingham	App
	Explicit Medication Tracker	A section named, "medication" or a "medication" tag that allows input of medication and dose	App
	Explicit Exercise Tracker	A section named, "exercise" or an "exercise" tag that allows input of medication and dose	App
	Explicit Diet – Salt Tracker	A section that has a reference to, "salt" or "sodium" that allows analysis of foods for salt or sodium content, or manually allows input of salt or sodium from foods in the diet	App
	Weight	Weight input in lbs or kg	App
	Explicit Symptoms Log	A section named, "symptoms" or a "symptoms" tag that allows for input of symptoms	App
	Referral of Patient to Legitimate Outside Resource for HTN Education	A clearly labeled external resource whose purpose is to educate the patient about HTN outside the app. The webpages are deemed by the investigators to be reliable and credible sources of information.	App
	Explicit Lab Values Tracker	A section named, "lab values", "lab", "biochemistry" or "values" or with corresponding tags that clearly allows input of values for lab values	App

Section	Subcategory	Description	Source
	Social Media Compatibility: Twitter, Facebook, Forums	Allows sharing of blood pressure readings with friends or family over Twitter, Facebook or other internet forums. It may also facilitate access to Facebook groups or forums about HTN.	App
**BP Tracking Features**	BP Goal Setting	Does the app allow the user to input a personalized goal BP (to the 1mmHg)	App
	Reminders	Does the app allow you to set reminders to take blood pressure readings?	App
	Sync BP data with BP monitor or cuff	Does the app automatically sync data with an external BP monitor	App
	BP log allows for at least 28 measurements in total, with duplicate measures QAM and QHS	The BP log allows for the recording of two distinct measurements in the morning and two distinct measurements in the evening of one 24h period. The BP log also allows recording of at least 28 measurements.	App
**Data Validation**	BP measurements have a maximum or minimum limit that you can input or flags improbably high/low readings or app flags inverted BP readings (DBP/SBP)	As part of the data input algorithm for each app, there are improbably high and low BP readings that should be flagged when input. If these are not flagged, the monitor does not have a system for identifying erroneous readings. As part of the data input algorithm for each app, there is a reading that is purposely inverted, that should be flagged when input. If this is not flagged, the monitor does not have a system for identifying erroneous readings. The investigator should determine the exact cutoffs for the readings (if present) and input those cutoffs into the Notes section.	App
	Pt can backdate BP measurements or specify exact time of day of BP reading	If the patient can go back in time to record a BP reading or specify the exact time of day (EX: 9:03AM) of a BP reading (instead of pre-set options like “AM” or “PM” only)	App
	Diary or personal notes for each measurement	Does the app allow the patient to record what was happening around the time of their BP reading (i.e., they drank coffee right before the reading, or they were stressed at work)	App
**BP Analytical Features**	BP Categories and Red Flags	Does the app have the correct normotensive, hypotensive, hypertensive categories and visually identify (or codify) when they have high, low or normal blood pressure readings	App
	BP Categories have References to Scientific Research	The BP categories the app uses explicitly references scientific research	App
	Suggests Appropriate course of action for Alert Ranges	If BP is too low or too high, the app tells the patient appropriate next steps, such as visiting their family physician or going to the ER	App

Section	Subcategory	Description	Source
	In-App Stat Analysis that includes at least the ability to calculate average BP	The app can, at minimum, calculate average BP for either pre-set dates or self-programmed dates. Other stat analyses could include IQR, minimum/maximum, median	App
	Export Data: Share collected data with other users or HCPs	The app allows users to share the collected BP measurements with other users or HCPs. Differentiate between sharing only one reading at a time, all readings at once, or a self-programmed number of readings.	App
	Live Sharing of Readings	The app automatically shares BP measurements or the app can sync BP measurements with a desktop program, HCPs or other users.	App
	Online Data Backup	The app allows users to back-up their BP measurements in the cloud	App
**Quality Assessment: HON Domains**	HCP Involvement	As noted in the Developer Website that a health care professional was directly involved in the creation of the app (not simply that there is a healthcare professional involved with the company).	Website
	Purpose/Disclaimer	The app states that its purpose is solely to record BP readings and is not a replacement for a HCP	App, App Store
	Privacy Policy - Present	There is a privacy policy specifically for the app, and not a generalized privacy policy for the developer website	App Store, App
	Privacy Policy - App shares information with 3rd party	The app states that it shares its information with a 3rd party, or it does not have a statement in the privacy policy that states it protects user data from being shared with a 3rd party	App Store, App
	Privacy Policy - Tracks information linked to patient	The app states that it tracks patient information, or it does not have a statement in the privacy policy that states it prevents user data from being tracked	App Store, App
	Support – contact address or feedback mechanisms for technical support	The app has a section for contacting the developer with questions about privacy, ethics or technical support	App Store, App
	Sponsored by …	The app is sponsored by or is developed by one of the listed organizations or device companies	App Store, App
	Conflict of interest: sponsorships are clearly labeled	The app clearly shows logos of sponsors or states their sponsors in the app itself	App Store, App
	Advertising present	Advertising is present in the app	App
	Advertising clearly marked and distinguishable from content	Advertising is clearly marked and distinguished from the app content	App
	Promotes a Specific Product	The app promotes the use of a specific product	App

Section	Subcategory	Description	Source
**Software Behavior**	Over testing period, # times the app has glitches or crashes	The number of times the app functions glitch or the app crashes over the testing period (app algorithm)	App
	Over testing period, there are functions that do not work	The number of functions the app has that are non-functional	App
**Quality of Education Material, if Present**<br><br>*Comprehensiveness of HTN Education (Present in its entirety: 2 Points, Some Points Present: 1 Point, No Points Present: 0 Points)*	Basic BP & HTN Information:	• Blood pressure is the force of blood against your blood vessels as it circulates through your body<br>• Hypertension is defined as blood pressure that is consistently above the normal range (>140/>90)<br>• Systolic pressure occurs when your heart contracts and is the higher of the two numbers. Diastolic pressure is the lower number and it occurs when your heart relaxes and fills with blood<br>• It is important to know your blood pressure<br>• High blood pressure does not usually have symptoms and many people do not know they have it<br>• High blood pressure increases risk of stroke, heart attack, heart failure, dementia, kidney disease, eye problems, sexual dysfunction<br><br>There are six listed facts that should be covered about HTN. For 1 mark to be awarded to the app for presenting a specific fact, all components of the each fact must be present in the app. (i.e., 0 marks for: systolic pressure is the higher number. 1 mark for: systolic is the higher number, and is when your heart contracts.)	App, Website
	Appropriate BP Measurement	• Wait 30 minutes after drinking coffee or smoking<br>• Rest quietly for 5 minutes before taking a measurement<br>• Sit with your feet flat on the floor, back and arm supported, and arm at heart level<br>• Put the cuff on a bare arm<br>• Consistently measure BP in R or L arm<br>• Checking your blood pressure regularly at home, and keeping a record, can be very useful<br>• Be sure to buy a blood pressure device that has the Hypertension Canada Approved logo<br><br>There are six listed facts that should be covered about appropriate BP measurement. For 1 mark to be awarded to the app for presenting a specific fact, all components of the each fact must be present in the app.	App, Website
	Nature of Treatment Options	• Smoking cessation<br>• Exercise<br>• Weight management<br>• Alcohol<br>• Relaxation techniques<br>• Dietary management	App, Website

Section	Subcategory	Description	Source
		The app should cover 6 topics for lifestyle management in patients with hypertension. These topics simply need to be listed, no details are required for this section of the criteria.	
	How to Use Treatment	• Take Medications as prescribed<br>• Some patients may need 2 or more medications<br><br>The app should have 2 statements regarding medications in HTN, and each statement must be covered in its entirety to award a mark for that particular statement.	App, Website
	Development of BP Self-Monitoring Skills	• Provides guidance on how to measure home BP using an automated device<br><br>The app should have some guidance on the technique of measuring BP using a home automated device	App, Website
	Personalized Action Plan:	BP Treatment goals for HTN patients is <135/85 for HBPM<br><br>The app should state the goal of treatment for home BP is <135/<85 mmHg	App, Website
	Recognition of Abnormal Values	1) Describes what to do with high BP readings and when to seek assistance.<br>2) Describes the signs and symptoms that require immediate attention<br><br>The app should describe what to do with a high BP reading, when patients need to seek assistance, and the signs and symptoms of cases that require an ER visit.	App, Website
	Links to a Health Care Provider	• Advocates discussion with HCP to set goals<br>• Advocates to show BP readings to HCP<br><br>The app should have 2 statements regarding discussing or sharing measurements with their HCP	App, Website
**Quality of Education Material, if Present** *(Each statement must be present in its entirety to award 1 point)*<br><br>*Consistency with Evidence-Based Guidelines: BP Measurement*	Appropriate BP Measurement<br>1) Wait 30 minutes after drinking coffee or smoking	If this statement is present in its entirety, a point is awarded	App, Website
	2) Rest quietly for 5 minutes before taking a measurement	If this statement is present in its entirety, a point is awarded	App, Website
	3) Sit with your feet flat on the floor, back and arm supported, and arm at heart level.	If this statement is present in its entirety, a point is awarded	App, Website

Section	Subcategory	Description	Source
	4) Put the cuff on a bare arm	If this statement is present in its entirety, a point is awarded	App, Website
	5) Consistently measure BP in R or L arm	If this statement is present in its entirety, a point is awarded	App, Website
	6) Be sure to buy a validated blood pressure device	If this statement is present in its entirety, a point is awarded	App, Website
	Home BP done at least twice QAM and QHS for at least 7 consecutive day	Statement that home BP monitoring must be measured twice QAM and twice QHS for at least 7 days consecutively	App, Website
	BP treatment goals for HTN pts is <135/85 for HBPM	Statement that home BP treatment goals are <135/85mmHg	App, Website
	Home BP monitor should be upper-arm automatic BP monitor with cuff that is appropriately fitted	Statement that patients should preferentially use an upper-arm automatic BP monitor with an appropriately fitted cuff	App, Website
**Quality of Education Material, if Present** *(Each statement must be present in its entirety to award 1 point)* *Consistency with Evidence-Based Guidelines: Lifestyle Management*	Advice to quit smoking should be provided	Information in the app advises smokers to quit smoking	App, Website
	30-60min of moderate intensity dynamic exercise 4-7d per week in addition to routine ADL	Statement that patients should exercise 30-60minutes daily for 4-7d in a week in addition to routine activities of daily living	App, Website
	BMI target 18.5 - 24.9 and WC <102 cm men, <88 cm women	Statement that BMI should be targeted within normal ranges, and waist circumference should be below the cutoff points	App, Website
	Alcohol restriction to ≤2 drinks per day (max 14 standard drinks for men, 9 standard drinks for women)	Statement that patients should restrict their alcohol intake to the stated cutoffs	App, Website
	Diet that emphasizes F/V, low fat dairy or DASH diet or Mediterranean diet	Statement that the diet should emphasize fruits and veggies, low fat dairy OR a statement containing a reference to the DASH diet OR a statement containing a reference to the Mediterranean diet	App, Website

Section	Subcategory	Description	Source
	Goal for sodium reduction to be between 2-5 g/d	Statement that patients should target sodium intake of 2g/d (of salt intake of 5g/day)	App, Website
Usability of Education Feature	Grammatical and spelling errors	The educational statements must have no grammatical or spelling errors	App, Website
	Written in plain language	The educational statements must be written at a level that would be understandable to the average reading level of an adult	App, Website
