# Supplementary material for: Quality of Blood Pressure Tracking Apps for the iPhone: Content Analysis and Evaluation of Adherence With Home Blood Pressure Measurement Best Practices
Source: JMIR Mhealth Uhealth. 2019 Apr 12;7(4):e10809. doi: 10.2196/10809 (PMC6484262; doi:10.2196/10809)
Supplement: Multimedia Appendix 2 [file mhealth_v7i4e10809_app2.pdf]

Multimedia Appendix 2. Mock Patient used for App Evaluation

Patient Profile
Name: Hyper Tension
Gender: Male
DOB: January 1, 1950
Blood pressure goal: 135/85, with reminders at 8AM and 5PM
Medications: Hydrochlorothiazide 25mg daily, Bisoprolol 5mg daily with reminders for both at 8AM daily
Weight: 100kg (220lbs)
Height: 168cm (5'6")
Symptoms: None
Physical activity: Walked 45minutes on Day 5, 2015 at 7 PM
Lab values: Serum Creatinine: 100 mmol/L

Date	Time	Blood Pressure (mmHg)		Heart Rate (bpm)
		Systolic	Diastolic	
Day 1	0800	90	70	30
		90	70	30
	1700	100	80	40
		100	80	40
Day 2	0800	110	90	50
		110	90	50
	1700	80	40	60
		80	40	60
Day 3	0800	130	110	70
		130	110	70
	1700	140	120	80
		140	120	80
Day 4*	0800	(15)*150	(3)*130	90
		150	130	90
	1700	180	100	100
		180	100	100
Day 5	0800	210	100	110
		210	100	110
	1700	90	70	120
		90	70	120
Day 6*	0800	(480)*180	(420)*120	50
		180	120	50
	1700	210	90	30
		210	90	30
Day 7*	0800	(80)*140	(140)*80	60
		140	80	60
	1700	100	60	40
		100	60	40

**Purposely created 3 readings with user errors that would only be corrected if the application flagged the reading. The first reading was an improbably low blood pressure and the second was an improbably high blood pressure that would indicate if there was a maximum or minimum limit that would trigger alarms by the HTN application. The third reading was inverted to test if the HTN application would automatically flag readings that were incorrectly input.*

Average SBP (discard first day): 143; Average DBP (discard first day): 93; Average HR (discard first day): 72
