# Supplementary material for: Quality of Blood Pressure Tracking Apps for the iPhone: Content Analysis and Evaluation of Adherence With Home Blood Pressure Measurement Best Practices
Source: JMIR Mhealth Uhealth. 2019 Apr 12;7(4):e10809. doi: 10.2196/10809 (PMC6484262; doi:10.2196/10809)
Supplement: Multimedia Appendix 3 [file mhealth_v7i4e10809_app3.pdf]

Multimedia Appendix 3. List of Included Apps

Name	Developer	Version	Updated
Braun Healthy Heart	Kaz Usa	1.0.3	Oct 31, 2015
Homedics	Homedics	2.4.3.2	Dec 2, 2016
BPMon - Blood Pressure Monitor	Anatoly Butko	10.1	Apr 17, 2017
Blood Pressure & Pulse Diary	Jan-Hendrick Damerau	2.2.1	Dec 20, 2016
Blood PressureDB - BP Blood Pressure Tracker	Klier.net	5.69	Mar 26, 2016
Blood Pressure Diary 2	cellhigh	1.7.1	Oct 8, 2016
HeartStar Blood Pressure Monitor	Pattern Health Techologies	3.2.0	Nov 28, 2016
Blood Pressure Companion Free	Maxwell Software	5.2.2	Jun 9, 2017
BP wiz - Blood Pressure Log and	Linklinks LTD	4.2.1	Jun 5, 2017
iBP Blood Pressure	Leading Edge Apps	9.0	May 16, 2016
MedM Blood Pressure	SwissMed Mobile AG	2.1.122	Apr 21, 2017
Blood Pressure Monitor- Family Lite	Taconic System LLC	3.3.2	May 10, 2017
VitaDock	Medisana AG	1.5.3	Nov 24, 2015
Blood Pressure - Smart Blood Pressure	Evolve Medical System	2.2	May 15, 2016
Cardio+	Microlife Corp	1.02	Dec 17, 2013
BP log - Blood Pressure Diary	ser.soft gmbh	1.4.1	Dec 2, 2016
Blood Pressure Passport Free	ecotouchmedia	1.4	Apr 24, 2015
bp Trax - Blood Pressure Treatment	imobilife	1.18	Jun 24, 2015
Blood pressure down	calories, LLC.	1.0	Mar 30, 2014
Blood Pressure Assistant - log	Josef moser	3.11	Jun 9, 2017
Blood Pressure Monitor Cloud Edition	Thomas Kress	1.0.7	Sep 24, 2017
Blood Pressure Tracker - Pro Version	iHealth Ventures LLC	2.8.0	Jul 19, 2014
blood pressure tracker quicklog	genehome	2.0.7	Jan 25, 2015
Vitals	Cory Bohon	1.0	May 26, 2016
IvyHealth	Ivy Health	1.3.3	Jun 9, 2017
Fast BP - Blood Pressure Log & Tracker	Christion Richert	1.2	Dec 18, 2015

Name	Developer	Version	Updated
Blood Pressure Lite - BP Tracker for Hypertension	Codulis	1.5.3	Mar 3, 2016
Free blood pressure monitor for iPhone	Dzmitry Permiakou	3.2.1	Apr 10, 2017
Monitor my BP by APG Solutions, LLC	APG Solutions	2.1.2	Dec 18, 2016
iTension	Les Laboratoires Servier	1.0.6	Sep 23, 2014
My blood Pressure readings	Puig Labs	1.92	Mar 31, 2016
Blood Pressure for Health	Angel Garcia Rubio	1.2.1	Nov 30, 2016
Easy blood pressure	Seaside Apps	2.3	Jan 14, 2016
Best blood pressure monitor	Andrei Mitrohin	1.2.5	Jul 20, 2015
Bloody Pressure	Piotr Sochalewski	2.3.1	Oct 30, 2016
BP Buddy - 80% off Sale - Blood Pressure	Azumio Inc.	1.4	Dec 9, 2007
Blood pressure Made Easy	Chad Dunlap	1.2	Sep 9, 2013
Goal Blood pressure	Benjamin Hysell	2.4.0	Feb 10, 2017
Easy BP	Moonstone Apps	1.24	Dec 17, 2012
Easy Blood Pressure Diary	SILECI	1.0.1	Jan 23, 2016
Blood Pressure Logger	Suprabhat chouksey	1.3	Mar 16, 2016
mybloodpressure	Arbitrary Software LLC	1.9	Feb 21, 2012
BPNote Lite	Hazelnutworks	1.2.10	May 11, 2017
BP recorder Free	Cappable Limited	2.7.1	Sep 20, 2016
Easy BP Life Log	Tetsuya Aihara	1.1.0	Apr 20, 2017
Your Blood Pressure	www machealth pty LTD	1.2	Aug 22, 2013
AGR Blood Pressure Log	Angel Garcia Rubio	1.3.4	Nov 30, 2016
Blood Pressure +Pulse Grapher Lite	Michael Heinz	1.5	May 24, 2017
Wellness Connected	A&D Medical	1.8.2	Dec 5, 2016
Heart Card	Kind of blue	1.5	May 4, 2017
BP Grapher	Kazuhiiko Kuroda	2.7	Apr 23, 2017

Name	Developer	Version	Updated
Chart my BP - Blood Pressure Tracker	Appstructure LLC	1.2	Nov 27, 2013
Simple Blood Pressure recorder	Stack3	2.0.2	May 20, 2017
iBP log	Jaab Productions	1.1	Oct 22, 2013
120 over 80	Jeffre Kempster	1.5	May 5, 2017
Bloodnote - blood pressure control	Matt Ludzen and Peter	2.0	Jan 23, 2015
Track my BP	New World Monkeys	2.0.1	Aug 21, 2015
bprecord	QuantumWorld	1.4	May 13, 2014
Blood Pressure tracker - By Japps	Japps	1.1	Aug 19, 2015
Health Graph	VVI	12.8.1	Jun 9, 2017
Blood Pressure Graphs	Shigeto Takagi	150509	May 18, 2015
Simple BP	Abhishek Karale	1.0	Mar 1, 2016
